# Prevention of Cystitis

**Published:** 1908-09

**Authors:** 


PREVENTION OF CYSTITIS.

Professor Howard A. Kelly in his "Medical Gynecology" (*Appleton & Co.*, New York, 1908), regarding the prevention of cystitis says: when the physician finds it is going to be necessary to catheterise his patient during convalescence, as after a severe confinement, one of the best prophylactic agents is the use of urotropin, say 10 grains thrice a day till danger of infection is over. Urotropin has its best field as a preventive in such cases and as an indispensable adjuvant in fresh infections. It is less effective in old cases. It seems to have more effect on the colon bacillus than on any other organism. It is useless in tuberculosis and probably most effective in cystitis and pyelitis following typhoid. In surgical cases prophylaxis can do a great deal to prevent cystitis following, and complicating convalescence.

In existing cystitis, urotropin (5 grains every 3 or 4 hours) should be given for some days at the beginning. If this makes the urine more irritating, the dose should be lessened or suspended.

